# Supplementary material for: Effect of neutering timing in relation to puberty on health in the female dog–a scoping review
Source: PLoS One. 2024 Oct 14;19(10):e0311779. doi: 10.1371/journal.pone.0311779 (PMC11472935; doi:10.1371/journal.pone.0311779)
Supplement: S1 File — The search terms used in a scoping review designed to identify and chart the current evidence on the effect of the timing of neutering in relation to puberty on health in female domesticated dogs. (DOCX) [file pone.0311779.s002.docx]

Supplementary material S1 Search terms

The search terms used in a scoping review designed to identify and chart the current evidence on the effect of the timing of neutering in relation to puberty on health in female domesticated dogs:

Neuter OR neuters OR neutering OR neutered OR spay OR spays OR spayed OR spaying OR ovariohysterectomy OR ovariohysterectomies OR sterilize OR sterilizes OR sterilization OR sterilise OR sterilises OR sterilisation OR gonadectomy OR gonadectomise OR gonadectomize OR ovariectomy OR ovariectomies OR de-sexing OR desex OR desexing OR de-sex OR castration OR castrate OR castrating OR sterilized OR sterilised

AND

Age OR pubert*

AND

Bitch OR bitches OR dog OR dogs OR canine OR canines OR canid OR canids OR canis

AND

Search 1

Obesity:

obese OR obesity OR “body fat” OR fatness OR adiposity OR overweight OR “over weight" OR grossness OR heaviness

Search 2

Neoplasia:

Neoplas* OR cancer* OR tumor* OR tumour* OR malignan* OR sarcoma* OR carcinoma* OR carcinosarcoma* OR haemangiosarcoma* OR hemangiosarcoma* OR osteosarcoma* OR lymphoma* OR lymphosarcoma* OR adenosarcoma* OR adenocarcinoma* OR lymphangiosarcoma* hemangiopericytoma* OR mastocytoma* OR mastocytosis* OR apudoma* OR mesothelioma OR lymphangiosarcoma OR myxosarcoma OR myosarcoma OR leiomyosarcoma OR fibrosarcoma OR chondrosarcoma OR liposarcoma OR hepatoblastoma OR mesenchymoma OR thymoma

Search 3

Urogenital:

Urinary tract infection* OR urinary incontinence OR urinary tract OR urologic* OR UTI* OR bladder OR cystitis OR vulva* OR vagina* OR vaginitis

Search 4

DOD:

“hip dysplasia” OR “elbow dysplasia” OR dysplastic OR coronoid OR “fragmented medial coronoid process” OR osteochondrosis OR osteochondritis OR “hypertrophic osteodystrophy” OR panosteitis OR “ununited anconeal process” OR “medial compartment disease” OR “short ulna syndrome” OR “trochlear notch incongruity” OR “short radius syndrome” OR “cruciate ligament” OR “cruciate rupture” OR cruciate OR “cruciate injury” OR “cruciate tear”

Search 5

Atopy:

exp Dermatitis, Atopic/

OR atopy OR “atopic dermatitis” OR dermatitis OR atopic OR eczema OR “allergic skin” OR pruriti* OR allerg* OR “allergic inhalant dermatitis”
