# Supplementary material for: Effect of neutering timing in relation to puberty on health in the female dog–a scoping review
Source: PLoS One. 2024 Oct 14;19(10):e0311779. doi: 10.1371/journal.pone.0311779 (PMC11472935; doi:10.1371/journal.pone.0311779)
Supplement: S2 File — The inclusion and exclusion criteria for a scoping review of the literature to identify and chart the current evidence on the effect of the timing of neutering in relation to puberty on the health of female domesticated dogs. (DOCX) [file pone.0311779.s003.docx]

Supplementary material S2 inclusion and exclusion criteria

The inclusion and exclusion criteria for a scoping review of the literature to identify and chart the current evidence on the effect of the timing of neutering in relation to puberty on the health of female domesticated dogs.

| Criteria | Inclusion | Exclusion |
| --- | --- | --- |
| POPULATION | Female domesticated dogs | Male dogs  Other species |
| Exposures | Entire female dogs presented for surgical neutering | Papers comparing entire bitches to neutered bitches  Papers not relating neutering to age or pubertal status  Papers relating to the treatment of dogs with behaviour problems |
| Intervention | Neutering procedure including chemical techniques and open and laparoscopic techniques which involve removal of the ovaries | Papers relating to neutering by hysterectomy |
| Outcome | Papers including assessments or measures of dog health  Owner observations or survey of dog health  Health to include obesity, neoplasia, developmental orthopaedic disease, urinary disease, atopic dermatitis, and vulval/vaginal disorders | Behavioural outcomes  Surgical outcomes in the  14-day perioperative period and behaviour relating to recovery following surgery  Pseudopregnancy  Health problems other than those specified  Student or owner opinions, perceptions or attitudes without health observation or measure  Papers only examining effect of neutering on control of reproduction |
| Language | English or papers with translation available | Translation not available |
| Study design | Case series, cohort, case control, cross sectional | Individual case reports, qualitative studies |
| Publication type | Peer reviewed journals  Conference proceedings | Narrative, textbook chapters, reviews (reference lists from systematic reviews were used to crosscheck we had all relevant studies but were not used themselves)  Unable to obtain full study details (abstract or full text)  Non-peer reviewed journals |
