# Supplementary material for: Effect of neutering timing in relation to puberty on health in the female dog–a scoping review
Source: PLoS One. 2024 Oct 14;19(10):e0311779. doi: 10.1371/journal.pone.0311779 (PMC11472935; doi:10.1371/journal.pone.0311779)
Supplement: S3 File — (DOCX) [file pone.0311779.s004.docx]

Supplementary material S3 - Scoping review extraction form

| Title |  |
| --- | --- |
| Authors |  |
| Year of publication |  |
| Source/country |  |
| Aims |  |
| Population and sample size  Breeds  Source – shelter / pet |  |
| Study design  Random allocation or retrospective ask when neutered  Consider health before neuter? |  |
| Excluded dogs neutered due to a pre-existing health problem |  |
| Methods |  |
| Intervention details |  |
| How define prepubertal |  |
| Did they actually note when bitch had first season |  |
| Did they note whether bitches had ever been bred? |  |
| Concept |  |
| Outcomes |  |
| How outcomes measured |  |
| Key findings that relate to review question |  |
