# Supplementary material for: Effect of neutering timing in relation to puberty on health in the female dog–a scoping review
Source: PLoS One. 2024 Oct 14;19(10):e0311779. doi: 10.1371/journal.pone.0311779 (PMC11472935; doi:10.1371/journal.pone.0311779)
Supplement: Attachment — Submitted filename: Response to reviewers.docx [file pone.0311779.s006.docx]

PONE-D-24-20315

Effect of neutering timing in relation to puberty on health in the female dog - a scoping review

Response to reviewers

Review Comments to the Author

Reviewer #1: In this scoping review, worthful details on studies dealing with the impact of neutering bitches before or after puberty on specific health issues are given, showing how difficult it is to compare these studies and that dogs were mostly not examined / puberty mostly not excluded. This is the highest benefit of this review. It is impressive that so far only 6 publications provided information about pubertal stage at neutering; in all others, neutering at a younger age may be more applicable. The provided scoping review appears technically sound and is presented in an intelligible fashion and standard English.

Response: Thank you very much for reviewing the manuscript and for your comments.

However, many of the here considered problems/diseases are multifactorial rendering reviews and especially meta-analyses very difficult as homogenous groups are difficult to find; neutering is probably only one factor leading to an aggravation of an already existing problem. I would really like to find a respective remark in the introduction, even though in the discussion it is well mentioned and elaborated.

Response: Thank you for your suggestion. The following sentence in the first paragraph of the introduction has been extended to read “However, comparing and consolidating evidence from different studies is challenging due to variations in study populations and methodological approaches, along with the multifactorial nature of the diseases studied.” We hope that this addition suitably acknowledges this point in the introduction.

Line 66: in the study of Schneider et al (1969), authors found a significant decrease in the relative risk of developing a mammary carcinoma, when female dogs were neutered before puberty. In most reviews I miss this clear differentiation between tumours/neoplasias in general and carcinomas. Even though the introduction shall only give an overview, it should be more differentiated.

Response: Thank you for identifying this oversight, and we agree that this is commonly not differentiated in many reviews. This sentence has been altered so that the cancers studied are clearly presented. This now reads “Most reviews reference the same work by Schneider *et al*. [20] that identified a reduced risk of histologically malignant mammary neoplasms (adenocarcinomas and mixed mammary tumours) for bitches neutered before the first oestrus.”

The discussion is somewhat lengthy; many details are provided in the tables that are very helpful. Maybe authors can shorten the text.

Response: Thank you for your suggestion. We have reviewed the discussion and we have removed a small amount of text and referred readers to Table 1 at one point. However, after reviewing the discussion, we think that the discussion relative to each category of disease, while for some categories is long, is useful to the reader to bring together a summary of the papers and related information in one place. For this reason, combined with the comments from the second reviewer, we decided to leave these sections of the discussion unchanged.

I really like the proposals what should be considered in future studies, at the end of the discussion. The conclusions are feasible.
Response: Thank you

Line 466: …that had

Response: This has been corrected, thank you

Reviewer #2: Dear authors thank you for providing this scoping review for the investigation of the effect of neutering of female dogs before and after puberty on several health outcomes. This review was a nicely- written and interesting study that failed to identify any evidence concerning the effect of neutering on atopic dermatitis, neoplasia, obesity, DOD and urogenital disease. As such it warrants publication in PLOS ONE.

Response: Thank you very much for reviewing the manuscript and for your comments.

My only comment was to change spey to spay in line 646.

Response: Thank you for the suggestion, however the misspelling was intentional. The term ‘spay’ was included in the search terms, however the term ‘spey’, was not. Therefore, no changes have been made and this remains in the limitations section of the manuscript.
